# Optimizing and Characterization of Soybean Oil Seed Cake Protein Hydrolysis: In Vitro Analysis

**DOI:** 10.1002/fsn3.70270

**Published:** 2025-05-19

**Authors:** Haifa Hamza, Deepika Kaushik, Harmandeep Kaur, Rajdeep Kaur, Matteo Bordiga, Yassine Jaouhari, Charalampos Proestos, Mukhtar Ahmed, Mohammad Rizwan Khan, Fatih Oz, Mukul Kumar

**Affiliations:** ^1^ Department of Food Technology and Nutrition Lovely Professional University Phagwara Punjab India; ^2^ Department of Biotechnology, Faculty of Applied Science and Biotechnology Shoolini University Solan Himachal Pradesh India; ^3^ Department of Pharmaceutical Sciences Università Degli Studi del Piemonte Orientale “A. Avogadro” Novara Italy; ^4^ Laboratory of Food Chemistry, Department of Chemistry, School of Sciences National and Kapodistrian University of Athens Zografou Athens Greece; ^5^ Department of Zoology, College of Science King Saud University Riyadh Saudi Arabia; ^6^ Department of Chemistry, College of Science King Saud University Riyadh Saudi Arabia; ^7^ Department of Food Engineering, Faculty of Agriculture Ataturk University Erzurum Turkey; ^8^ East Anatolian High Technology Research and Application Center (DAYTAM) Ataturk University Erzurum Turkey; ^9^ Engineering Faculty, Department of Food Engineering Kyrgyz‐Turkish Manas University Bishkek Kyrgyzstan

**Keywords:** enzyme, functional properties, oil seed cake, protein hydrolysate, soybean

## Abstract

The study aimed to optimize the hydrolysis process of soybean oil seeds to produce protein hydrolysate powder with enhanced functional properties. The proximate analysis revealed that the hydrolysate had a significantly higher protein content (60.33%) compared to the original oil seed cake (46.26%). Using response surface methodology, the optimum condition of hydrolysis was found to be at pH 8 with an enzyme concentration of 0.3%. Under these conditions, the hydrolysate showed high antioxidant activity of (45.80%), total phenolic content of (1.80 mg GAE/g), and total flavonoid content of (0.54 mg QE/g). The techno‐functional properties of the optimized hydrolysate powder, including bulk density (0.51 g/mL), tapped density (0.66 g/mL), foaming capacity (22%), foam stability (50.4%), water absorption index (2.28 g/g), water solubility (59.66%), and oil absorption capacity (1.34 g/g) were found to be superior compared to other protein hydrolysates from literature. Characterization by FTIR revealed the presence of alcohol, alkane, amine, fluoro, and halo compounds, while XRD indicated a semi‐crystalline nature. SEM analysis showed a microporous, broken, and brittle morphology. The hydrolysate also exhibited promising bioactivities, with 40.33% lipase inhibition, 53.47% amylase inhibition, and prolonged glucose retention time up to 240 min in an in vitro model. These results demonstrate the potential of the optimized soybean oil seed cake protein hydrolysate as a value‐added product for applications in food, pharmaceutical, and biotechnology industries.

## Graphical abstract

This study focused on optimizing the hydrolysis of soybean oil seeds to create a protein hydrolysate powder with improved functional properties. The resulting hydrolysate showed a significantly higher protein content and, under optimized conditions (pH 8, 0.3% enzyme), exhibited enhanced antioxidant activity, phenolic content, and flavonoid content. Furthermore, the optimized hydrolysate powder displayed superior techno‐functional properties and promising bioactivities, suggesting its potential for various industrial applications.

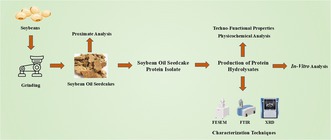

**Abbreviations**
AC: antioxidant capacities
CCD: central composite design
DH: degree of hydrolysis
FE‐SEM: Field Emission Scanning Electron Microscopy
FTIR: Fourier Transform Infrared Spectroscopy
OAC: oil absorption index
OCs: oilseed cakes
RSM: response surface methodology
SD: standard deviations
SEM: standard error of the mean
SPI: soy protein isolate
TFC: total flavonoid content
TPC: total phenolic content
WAI: water absorption index
WSI: water solubility index
XRD: x‐ray diffraction

## 1 Introduction

Oilseeds are significant plant‐based resources, distinguished by their high content of essential oils, dietary lipids, proteins, fibers, vitamins, antioxidants, and bioactive compounds. The byproducts of oilseed processing, particularly oilseed cakes (OCs), have gained increasing interest for their potential to develop value‐added functional foods and other products through innovative technologies (Usman et al. 2023). These OCs have become popular in animal feed formulations due to their cost‐effectiveness and nutritional profile (Abedini et al. 2022).

Soybean, sunflower, rapeseed, coconut, peanut, palm kernel, cottonseed, sesame seeds, and linseeds are the most popular oilseeds used in animal feed cakes (Rani and Badwaik 2021). Among oilseeds, soybean (*Glycine max L. Merril*) stands out as the most important seed legume globally, contributing significantly to edible oil production and protein concentrate for livestock feed (Skøt et al. 2023). Soybean oil seed cake, a byproduct of oil extraction, is widely utilized in animal nutrition owing to its high protein content and well‐balanced amino acid composition. It is the standard against which other protein supplements are compared (Arrutia et al. 2020) The defatted soybean meal obtained by extraction of soybean oil is also used to produce soybean protein isolate (SPI) which is a hydrolysate. The characteristic beany flavor of soybeans is still present in the items made from soybean meal, significantly lowering their flavor quality.

Protein hydrolysates are derived from protein sources through thermal processes combined with acid treatment or enzymatic hydrolysis. These hydrolysates comprise a mixture of peptides of varying lengths and free amino acids (Ulug et al. 2021). The degree of hydrolysis (DH) characterizes these products, indicating the extent of protein breakdown (Arrutia et al. 2020). Protein hydrolysates exhibit advantageous functional properties and find applications in human and animal nutrition, with potential health benefits such as blood pressure regulation (Miguel et al. 2020). Enzymatic hydrolysis has proven effective in enhancing the functionality and utilization of soybean meal. Alkaline proteases (EC.3.4.21–24, 99), which operate in neutral to alkaline pH ranges, are widely used in various industries, including food processing, pharmaceuticals, and detergent manufacturing (Pawar et al. 2023). The enzymatic hydrolysis of oilseed cake proteins results in improved solubility, emulsification properties, and digestibility, while reducing bitterness (Subaşı et al. 2021). Soybean oil seed cake is a low‐valued byproduct as processing these byproducts into more valuable forms can have a substantial economic and environmental impact; however, the production of protein hydrolysate from it can be an opportunity to convert it into a valuable resource. These hydrolysates have potential applications in vaccine production, animal nutrition, and as plant growth regulators (Shahrajabian et al. 2022; Colletti et al. 2020). Utilizing alkaline protease enzymes for hydrolysis can unlock the full potential of this byproduct, contributing to sustainable resource management practices. By repurposing this underutilized byproduct, we seek to contribute to the development of sustainable and value‐added products from agricultural waste streams. The outcomes of this study will provide valuable insights into the efficient utilization of soybean oil seed cake, potentially leading to the development of novel protein hydrolysates with enhanced functional and nutritional properties. Future applications for soybean oil seed cake‐derived protein hydrolysates include their use as functional ingredients in food and beverages, nutraceuticals, and pharmaceuticals. There is also growing interest in their potential as a sustainable alternative in animal feed and crop cultivation. Additional areas of exploration include cosmetics, personal care products, and bioremediation.

This paper aims to investigate the production of protein hydrolysates from soybean oil seed cake using alkaline protease enzymes. The study will focus on optimizing the hydrolysis process using RSM, characterizing the resulting products using FTIR, SEM, XRD, and analyzing their anti‐obesity and anti‐diabetic properties, thus evaluating their potential applications across various industries.

## 2 Materials and Methods

### 2.1 Materials

Deoiled soybean oil seed cakes were collected from Shazam farms, Hyderabad, Telangana, in the months of January–February 2024. Alkaline protease enzymes were collected from Enzyme Bioscience Private Limited, Gujarat. Fortune Soybean Edible Oil was collected from the local market of Phagwara. Sulfuric acid, sodium hydroxide, ethanol, HCl, NaOH, DPPH, sodium acetate buffer, methanol, quercetin, sodium nitrite, aluminum chloride, Folin–Ciocalteu reagent, gallic acid, TES buffer, lecithin, sodium cholate, glycerol trioleate, lipase, soluble starch, acetate buffer, distilled water, α‐amylase solution, dialysis membrane (MWCO: 2000), NaCl, glucose, potassium sulfate, mixed indicator, copper sulfate, boric acid were procured from Hi‐Media Chemicals Pvt. Limited (Mumbai, India). Analytical grade chemicals and Class “A” certified glassware, cleaned with triple distilled water, were used throughout the experiments.

### 2.2 Methods

#### 2.2.1 Grinding and Filtration

The collected soybean oil seed cake was ground with the help of a grinder (Sujata Powermatic plus 900 watts juicer mixer grinder) and filtered using a 300 μm sieve with the use of an electric sieve shaker manufactured by Bionics Scientific Technologies (P) Ltd., Delhi, India. The powder was then filled into plastic bags (Ziploc) and kept in a locker at room temperature to avoid moisture and contamination.

#### 2.2.2 Proximate Analysis

##### 2.2.2.1 Moisture Content

Five grams of powdered soybean oil seed cake sample was weighed and placed on a pre‐weighed petri plate. The Petri plate was then placed in an oven manufactured by Bio Technics India, Mumbai, India at 105°C for 7 h till it attained a constant weight (Jaglan et al. 2023). Moisture content was then calculated with the following formula:

(1) $$\text{Moisture}\;\left(\%\right)=\frac{\left(W1-W2\right)}{\mathrm{WS}}\times 100$$

where, W1 = initial weight of petri plate with sample (g).

W2 = final weight of petri plate with sample after drying (g).

WS = weight of the sample (g).

##### 2.2.2.2 Ash Content Determination

Two grams of soybean oil seed cake powder was taken in a pre‐weighed crucible, and charring was done. The crucible was then placed into the muffle furnace (manufactured by Stericox, Delhi, India) at 600°C for 2 h, followed by cooling in a desiccator and then the weight was measured. (Jaglan et al. 2023). Ash content was calculated with the following formula:

(2) $$\mathrm{Ash}\left(\%\right)=\frac{\left(W1-W2\right)\;}{\mathrm{WS}}\times 100$$

where, W1 = weight of crucible + sample after dry ashing (g).

W2 = weight of empty crucible (g).

WS = weight of the sample (g).

##### 2.2.2.3 Crude Fat Determination

The crude fat of soybean oil seed cake powder was determined using the Soxhlet apparatus. Three grams of powdered sample was taken into a thimble, and the extraction was done using petroleum ether as a solvent and heated at 60°C for 8 h in a Soxhlet extractor. The solvent can then be removed below the boiling point of petroleum ether by reducing the pressure with a vacuum, which lowers its boiling point and allows evaporation at lower temperatures. It is usually done by a rotary evaporator or gently heating under reduced pressure. The difference in the initial weight and the final weight of the flask was recorded (Jaglan et al. 2023).

(3) $$\text{Crude}\;\mathrm{fat}\left(\%\right)=\frac{\left(W2–W1\right)\;}{\mathrm{WS}}\times 100$$

where, W1 = initial weight of empty flask (g).

W2 = weight of flask after evaporation (g).

WS = weight of the sample (g).

##### 2.2.2.4 Crude Fiber Determination

A two‐gram sample of defatted soybean oil seed cake underwent acid–base digestion. It was first boiled in 200 mL sulfuric acid, then in 200 mL sodium hydroxide, with filtration and washing steps between. The residue was then washed with dilute 1.25% sodium hydroxide, water, and ethanol. Finally, it was dried, weighed, ignited at 600°C ± 15°C for 30 min, high temperature, cooled, and weighed (Jaglan et al. 2023).

(4) $$\text{Crude fiber}\;\left(\%\right)=\frac{\text{Weight of residue}–\text{Weight of}\;\mathrm{ash}}{\text{Weight of the sample}}\times 100$$

##### 2.2.2.5 Protein Content Determination

The protein content of oil seed cake powder was determined by a method prescribed by Jaglan et al. (2023) using the Kjeldahl method with slight modifications in digestion and distillation. The protein percentage was calculated using the formula:

(5) $$\text{Nitrogen}\left(\%\right)=\frac{14.01\times 0.1N\times \left(\mathrm{TV}-\mathrm{BV}\right)\times 100}{\mathrm{WS}\times 1000}$$

where, 0.1 = normality of H_2_SO_4_.

14.01 = molecular weight of ammonia.

TV = titer value.

BV = blank value.

WS = weight of sample

(6) $$\text{Protein}\;\left(\%\right)=\text{Nitrogen}\;\left(\%\right)\times 6.25$$

##### 2.2.2.6 Carbohydrate Content Determination

Total carbohydrate was calculated by using the methods prescribed by (Jaiswal and Shankar 2022). Moreover, the body is unable to digest fiber, a kind of carbohydrate. Although the majority of carbs are converted into glucose, which is a sugar molecule, fiber cannot be converted into glucose and instead moves through the body undigested (The Nutrition Source 2022). The carbohydrate percentage was calculated using the formula:

(7) $$\text{Carbohydrate}\;\left(\%\right)=100-\left(\mathrm{Fat}+\text{Moisture}+\mathrm{Ash}+\text{Fiber}+\text{Protein}\right)$$

#### 2.2.3 Isolation of Soybean Oil Seed Cake Protein

Oil seed cake was suspended in water (1:10 *w*/*v*) at room temperature, pH 8, and stirred for 1 h. The fiber was removed by centrifugation (42,205× *g*, 5°C) with the help of a centrifuge machine manufactured by Nes India Engineers, Pune, India. The supernatant was acidified to pH 4.5 with 2 M HCl and centrifuged after 2 h at 4°C. The resulting precipitate was washed with sodium acetate buffer (10 mM, pH 4.5, 1:8 *w*/*v*) and then re‐centrifuged. The final precipitate (SPI) was adjusted to pH 7.0 and dried (Sami 2017).

#### 2.2.4 Optimizing the Conditions for Hydrolysis of Soybean Oil Seed Cake Protein Isolate

The protein isolate is further utilized for preparing protein hydrolysate. Response Surface Methodology (RSM) software using central composite design (CCD) was employed to optimize the hydrolysis conditions for soybean oil seed cake protein isolate for producing the protein hydrolysate. RSM facilitates the design of experiments, generates mathematical models, and identifies the optimal combination of variables (Veza et al. 2023; Amiri et al. 2024). Table 1 shows the runs performed in RSM for the optimization of hydrolysis conditions. The total phenolic content (TPC), total flavonoid content (TFC), and antioxidant content were analyzed for each experimental run. The set of conditions that yielded the best values for these parameters was selected as the optimal conditions for hydrolyzing the soybean oil seed cake protein isolate.

**TABLE 1:** Experimental data for pH and Enzyme concentration (%) using response surface methodology analysis to optimize the conditions for hydrolysis of soybean oil seed cake protein isolate.

Run	Factor 1	Factor 2
	pH	Enzyme concentration (%)
1	10.40	0.65
2	8.00	0.30
3	9.00	0.15
4	10.00	0.30
5	10.00	1.00
6	9.00	1.14
7	9.00	0.65
8	8.00	1.00
9	9.00	0.65
10	9.00	0.65
11	9.00	0.65
12	9.00	0.65
13	7.58	0.65

#### 2.2.5 Production of Protein Hydrolysates From Isolated Proteins

Isolated soybean oil seed cake protein (5% *w*/*v* in water) was homogenized, pre‐incubated, then hydrolyzed with alkaline protease (pH 8, 45°C, 4.5 h). The enzyme was heat‐deactivated at 98°C for 10 min. Centrifugation separated soluble and insoluble fractions. The supernatant was lyophilized to produce the hydrolysate (Sami 2017).

#### 2.2.6 Antioxidant Capacity With the DPPH

The antioxidant activity of the hydrolysate extract was determined by a procedure given by Malik et al. (2021) with slight modifications. To conduct the analysis, 0.1 mL of hydrolysate extract, each with a concentration of, was combined with 3.9 mL of 0.1 mM DPPH solution and thoroughly mixed. The resulting mixture was allowed to react for 45 min, after which the absorbance of the solution was measured at 517 nm using a spectrophotometer manufactured by Linco Scientific Instrument & Chemicals Pvt. Ltd., Ambala, India. The antioxidant capacities (AC) of the extracts were expressed according to the formula:

(8) $$\text{Antioxidant Capacity}\;\left(\%\right)=\left(1-\frac{A}{A_{0}}\right)\times 100$$

where, *A* = absorbance of the sample.

*A*_0_ = absorbance of the control.

#### 2.2.7 Total Flavonoid Content

Using a slightly modified version of Zulkifli et al. (2020), the flavonoid content of the sample extract was determined using spectrophotometry. In 10 mL of methanol, dissolve 0.1 g of dry material. A quercetin standard curve was constructed. In a 10 mL volumetric flask, combine 0.3 mL of 5% sodium nitrite, 4 mL of water, and 1 mL of sample solution. After 5 min, add 0.3 mL of 2% aluminum chloride. Add 2 mL of 1 M sodium hydroxide after 6 min of waiting. Dilute with water at 510 nm to get an absorbance range of 0.1 to 1. Calculate the total quantity of flavonoids by comparing the sample's absorbance to a methanol blank. The standard calibration curve was generated by repeating the experiment with the quercetin standard solution. The quercetin calibration curve was used to calculate TFC, which was then expressed as mg quercetin equivalent (mg QE/g of extract).

#### 2.2.8 Total Phenolic Content

To determine the total phenolic content, combine 0.1 g of sample with 10 mL of ethanol, then add 250 μL of FC reagent, 100 μL of sodium carbonate (7.5%) solution, and incubate it for 30 min at room temperature. Use a spectrophotometer to measure the absorbance at 765 nm. To determine the total phenolic content, compare the sample's absorbance to a standard curve for gallic acid (Kumar et al. 2023).

#### 2.2.9 Techno‐Functional Properties

##### 2.2.9.1 Bulk Density and Tapped Density

Bulk density and tapped density were determined by pouring a known mass of powder into a graduated cylinder to measure bulk density and subjecting the powder to tapping to measure tapped density (Rani and Badwaik 2021).

(9) $$\text{Bulk density}=\frac{\left(\text{Weight of container}+\text{Sample}\right)–\text{Weight of container}}{\text{Volume of the container}}$$

(10) $$\mathrm{Tap}\;\text{density}=\frac{\left(\text{Weight of container}+\text{Sample}\right)–\text{Weight of container}}{\text{Volume of the container}}$$

##### 2.2.9.2 Foam Capacity and Foam Stability

A 100 mL of distilled water containing 0.5 g of the sample was placed in a 250 mL beaker. The solution was stirred using a magnetic stirrer set at 3798 g force for 5 min. After stirring, the solution was promptly transferred to a 250 mL graduated cylinder, and measurements were taken to determine the foam capacity. Foam stability was assessed by measuring the remaining volume after 3 min at 20°C (Chawla and Bains 2020; Li et al. 2024).

(11) $$\mathrm{FC}\left(\%\right)=\frac{\text{Volume after agitation}–\text{Volume prior to agitation}}{\text{Volume prior to agitation}\;}\times 100$$

(12) $$\mathrm{FS}\left(\%\right)=\frac{\text{Residual foam volume}}{\text{Total foam volume}\;}\times 100$$

##### 2.2.9.3 Water Absorption Index

The hydrolysate powder was mixed with distilled water and stirred gently for 30 min at room temperature. Afterward, the mixture was centrifuged at 15194 g force for 15 min with the help of a centrifuge machine manufactured by Nes India Engineers, Pune, India. The liquid portion was then transferred to a pre‐weighed Petriplate dish, while the remaining gel was weighed separately. The Water Absorption Index (WAI) was determined as the ratio of the weight of gel obtained to the weight of the solid material used (Patil et al. 2023).

(13) $$\mathrm{WAI}=\frac{\text{Weight of sediment}\;}{\text{Weight of}\;\mathrm{dry}\;\text{solid}}$$

##### 2.2.9.4 Water Solubility Index

The Water solubility index (WSI) was the weight of dry solids in the supernatant from the water absorption index test expressed as a percentage of the original weight of the sample (Yousf et al. 2017).

(14) $$\mathrm{WSI}\;\left(\%\right)=\frac{\text{Weight of dissolved solid in supernatant}}{\text{Weight of}\;\mathrm{dry}\;\text{solids}\;}\times 100$$

##### 2.2.9.5 Oil Absorption Capacity (OAC)

The 500 mg of sample was mixed with 10 mL sunflower oil and vortexed (10 min, 25°C) followed by centrifugation (4500 g, 30 min, 25°C). Unbound oil was decanted, and absorption was calculated by weight difference (Patil et al. 2023).

#### 2.2.10 Characterization Techniques

##### 2.2.10.1 Fourier Transform Infrared Spectroscopy (FTIR)

FTIR of the hydrolysate was performed on the Perkin Elmer Spectrum IR Version 10.6.1 with ATR and Pallet accessories. The data were analyzed with the help of spectrum software (Systat). For analysis, the sample was taken, and the spectra results were recorded at the mid‐infrared region (4000–400 cm^−1^) (Jozanikohan and Abarghooei 2022).

##### 2.2.10.2 Field Emission Scanning Electron Microscopy (FE‐SEM)

FESEM stands as the most adaptable method for examining the surface structure of a sample. The analysis of the protein hydrolysate was done using FE‐SEM coupled with an EDS detector; Au Sputter Coater (JEOL JSM‐7610F Plus, EDS: OXFORD EDS LN2 Free, Au Coater: JEOL Smart Coater). It was used for observing largely magnified images by using electrons instead of light to form an image at 100×, 1500×, and 10,000× (Zhang et al. 2021).

##### 2.2.10.3 X‐Ray Diffraction (XRD)

XRD is utilized to determine the crystal structure and composition of materials by analyzing their X‐ray diffraction patterns. XRD Bruker D8 Advance was used for the analysis of the protein hydrolysate. Each protein powder sample was scanned in a continuous mode at a scanning speed rate of 5°/min with the diffraction angle in the 2θ from 5° to 80° (Noman et al. 2020).

#### 2.2.11 Lipase Inhibition Assay

Lipase inhibition was assessed using Kumar et al.'s (2020) method. A substrate solution was prepared in TES buffer (pH 7.0) containing lecithin, sodium cholate, and glycerol trioleate. Sample extracts were mixed with substrate solution, lipase solution, and then incubated at 37°C for 30 min. Absorbance was measured at 550 nm. Lipase inhibitory activity (%) was calculated using the formula:

(15) $$\text{Lipase Inhibition}\%=1-\frac{\left(\mathrm{OD}2-\mathrm{OD}1\right)}{\left(\mathrm{OD}4-\mathrm{OD}3\right)}\times 100$$

where, OD1 = optical density of solution containing sample lipase, extract, and substrate.

OD2 = optical density of solution containing sample extract and substrate.

OD3 = optical density of incubated containing lipase and substrate.

OD4 = optical density of a solution containing substrate.

#### 2.2.12 Amylase Inhibition Assay

With slight modification from Kumar et al. (2020), the starch substrate (pH 7.0) was mixed with sample extracts in acetate buffer (pH 6.5), α‐amylase solution, and incubated (25°C, 15 min). The reaction stopped with HCl, and then iodine solution was added. Absorbance was measured at 650 nm using a Visible Spectrophotometer.

(16) $$\text{Amylase inhibitory activity}\%=1-\frac{\left(\mathrm{ODb}-\mathrm{ODa}\right)}{\left(\mathrm{ODd}-\mathrm{ODc}\right)}\times 100$$

where, ODa = optical density of solution containing amylase, sample extract, and starch.

ODb = optical density of the solution containing the sample extract and starch.

ODc = optical density of solution containing amylase and starch.

ODd = optical density of the solution containing starch.

#### 2.2.13 Glucose Uptake Assays Using Dialysis Membrane

Glucose movement was assessed using a 2000 kDa dialysis membrane. Extract (1 g/10 mL) plus glucose solution (15 mL, 0.22 mM) were mixed in the membrane and incubated (37°C, 4 h) followed by Centrifugation. Then, NaCl solution was added and Glucose diffusion was measured at intervals (15–480 min) using a glucometer manufactured by Sai Scientific & Surgical, Thane, India (Kumar et al. 2020).

#### 2.2.14 Statistical Analysis

Analyses were performed in triplicate, with results presented as mean values ± standard deviations (SD). Microsoft Excel 2016, Microsoft Corporation, Redmond, Washington, USA was used to calculate Standard Error of the Mean (SEM). One‐Way ANOVA tests identified differences among samples, determining critical differences in means and variance (Rani and Badwaik 2021). Hydrolysis conditions were optimized using RSM with Design‐Expert software (Version 8.0.11), State Ease, Minneapolis, Minnesota, USA. A full factorial Central Composite Design was employed for two independent variables: pH (*X*_1_) and enzyme concentration (*X*_2_). Coefficients were derived through multiple regression analysis. The experimental data obtained from CCD were subjected to response surface regression analysis and fitted to a second‐order polynomial model:

(17) $$Y=\beta_{0}+\beta_{1}X_{1}+\beta_{2}X_{2}+\beta_{11}X_{211}+\beta_{22}X_{22}+\beta_{12}X_{1}X_{2}$$

where *Y* is the response (dependent variable), *X*_1_ and *X*_2_ are the independent variables (pH and enzyme concentration), *β*_0_ is the intercept term, *β*_1_ and *β*_2_ are the linear coefficients, *β*_11_ and *β*_22_ are the quadratic coefficients, and *β*_12_ is the interaction coefficient (Roslan et al. 2014).

## 3 Results and Discussion

### 3.1 Proximate Composition of Soybean Oil Seed Cake and Soybean Oil Seed Cake Hydrolysate

**FIGURE 1:**
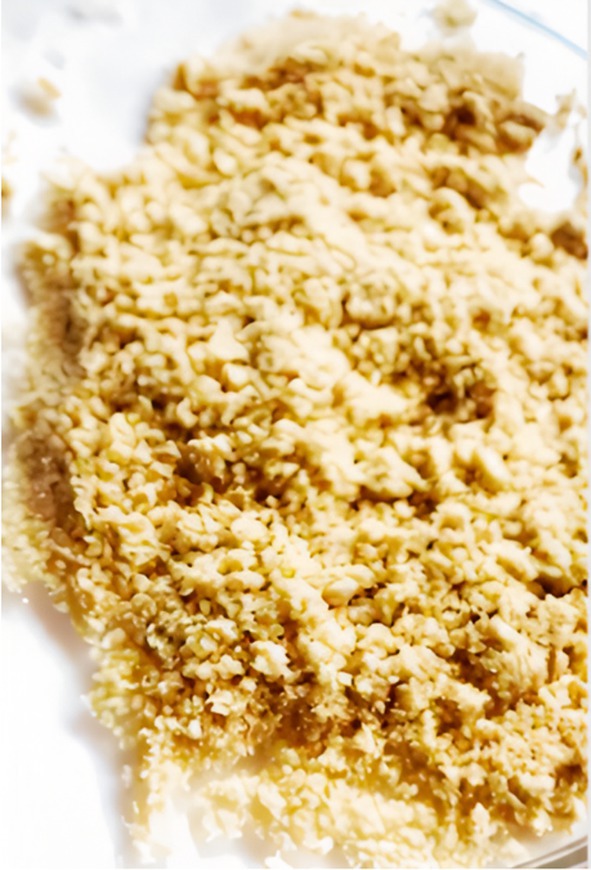
Soybean oil seed cake protein hydrolysate powder.

The proximate analysis of soybean oil seed cake and its protein hydrolysate is shown in Table 2, and Figure 1 represents the soybean oil seed cake hydrolysate. The soybean oil seed cake contained 11.76% moisture, 7.55% ash, 6.56% fat, 7.50% crude fiber, 46.26% protein, and 20.39% carbohydrates, indicating it is a rich source of protein and fiber. The results were compared with the results reported by Rani and Badwaik (2021) in which moisture content was 9.6%, ash 7.53%, fat 1.20%, crude fiber 45.83%, protein 44.47%, and carbohydrate 30.2%. Proximate analysis by Hallouch et al. (2024) also showed some difference, and the results were such as moisture content 5.54%, ash 5.78%, fat 1.25%, protein 44.85%, and carbohydrate 42.58%. The significant differences could be due to varietal variations, growing conditions, processing methods, oil extraction efficiency, and storage conditions (Arrutia et al. 2020).

**TABLE 2:** Proximate composition of soybean oilseed cake and soybean oilseed cake protein hydrolysate.

Parameters (%)	Soybean oil seed cake	Soybean oil seed cake protein hydrolysate
Moisture	11.76 ± 0.15^a^	5.16 ± 0.15^b^
Fat	6.56 ± 0.15^a^	4.76 ± 0.25^b^
Crude fiber	7.50 ± 0.10^a^	3.10 ± 0.10^b^
Protein	46.26 ± 0.80^b^	60.33 ± 1.52^a^
Ash	7.53 ± 0.20^a^	7.26 ± 0.30^a^
Carbohydrate (by difference)	20.39 ± 0.53^a^	19.39 ± 0.53^a^

*Note:* Data are presented as mean ± SD (*n* = 3); ^a,b^means with the same superscript in a row do not vary significantly (*p* < 0.05) from each other.

The soybean oil seed cake protein hydrolysate powder had an even higher protein content of 60.33%, along with 5.16% moisture, 7.26% ash, 4.46% fat, 3.10% crude fiber, and 19.39% carbohydrates. These findings aligned with other studies on protein hydrolysates from soybean and Jatropha cake by researchers like Apiwatanapiwat et al. (2009), Islam et al. (2022), which also reported high protein levels ranging from 50.21% to 71.69%. Protein hydrolysates have increased bioavailability and are valuable for functional food applications due to the enzymatic hydrolysis process that selectively cleaves peptide bonds, yielding a mixture of smaller peptides and amino acids without significant degradation of the protein material, as explained by Lafarga and Hayes (2016).

### 3.2 Optimizing the Conditions for Hydrolysis of Soybean Oil Seed Cake Protein Isolate

Methodology of the response surface using central composite design was employed to optimize the conditions for hydrolyzing soybean oil seed cake isolate. The pH and enzyme concentration were the specific parameters being studied, with each factor being measured at three different levels.

The response surface model was as follows:

(18) $$\begin{array}{c} \mathrm{TPC}\;\left(\mathrm{mg}\;\mathrm{GAE}/g\right)=+1.17-0.1081\;\left(A\right)-0.3469\;\left(B\right)\\ +0.6804\;\left(\mathrm{AB}\right)+0.4128\;\left(A^{2}\right)+0.4630\;\left(B^{2}\right) \end{array}$$

(19) $$\begin{array}{c} \mathrm{TFC}\;\left(\mathrm{mg}\;\mathrm{QE}/g\right)=+0.3964-0.0052\;\left(A\right)-0.0552\;\left(B\right)\\ +0.1393\;\left(\mathrm{AB}\right)+0.0846\;\left(A^{2}\right)+0.0809\;\left(B^{2}\right) \end{array}$$

(20) $$\begin{array}{c} \text{Antioxidant activity}\;\left(\%\right)=+37.06+5.43\;\left(A\right)-9.58\;\left(B\right)\\ -3.12\;\left(\mathrm{AB}\right)-4.83\;\left(A^{2}\right)-2.72\;\left(B^{2}\right) \end{array}$$

where, A is the pH, B is the enzyme concentration in wt %.

Table 3 shows that different treatments with varying pH levels and enzyme concentrations produce numerous results in terms of TPC, TFC, and antioxidant activity. The TPC of 1.80 mg GAE/g with a predicted value of 1.88 mg GAE/g, the TFC of 0.54 mg QE/g with a predicted value of 0.51 mg QE/g, and the antioxidant activity of 45.80% with a predicted value of 44.85% were measured in treatment 2, which was considered overall the best condition for hydrolysis. According to Zaky et al. (2019), a similar result for the TPC content was found to be 2.32 mg GAE/g for rice bran protein hydrolysates. Goswami et al. (2021) found that the mushroom protein hydrolysate had a TFC value of 0.47 mg CAE/g, which is similar to the TFC value of the optimized hydrolysate. Comparable results for antioxidant activity were observed in a study by Zhang et al. (2015), where soya protein hydrolysates had an antioxidant percentage of 51.7%.

**TABLE 3:** Experimental setup and results for the central design matrix.

Sr. No.	pH	Enzyme concentration (%)	TPC (mgGAE/g)	TFC (mgQE/g)	Antioxidant activity (%)
1	10.40	0.65	1.78 ± 0.08^b^	0.53 ± 0.04^ab^	35.09 ± 0.2^f^
2	8.00	0.30	1.80 ± 0.08^ab^	0.54 ± 0.05^a^	45.80 ± 0.4^c^
3	9.00	0.15	1.87 ± 0.02^ab^	0.48 ± 0.06^bc^	51.13 ± 0.19^a^
4	10.00	0.30	1.37 ± 0.01^e^	0.46 ± 0.01^cd^	46.83 ± 0.3^b^
5	10.00	1.00	1.89 ± 0.02^a^	0.57 ± 0.04^a^	27.06 ± 0.23^h^
6	9.00	1.14	1.54 ± 0.02^cd^	0.47 ± 0.03^cd^	23.09 ± 0.21^i^
7	9.00	0.65	1.64 ± 0.08^c^	0.42 ± 0.04^cde^	35.73 ± 0.74^f^
8	8.00	1.00	1.05 ± 0.02^f^	0.39 ± 0.04^e^	27.05 ± 0.55^h^
9	9.00	0.65	1.07 ± 0.05^f^	0.41 ± 0.01^de^	36.88 ± 0.20^e^
10	9.00	0.65	1.08 ± 0.03^f^	0.38 ± 0.05^e^	37.04 ± 0.55^e^
11	9.00	0.65	1.04 ± 0.08^f^	0.39 ± 0.02^cde^	37.85 ± 0.55^d^
12	9.00	0.65	1.09 ± 0.07^f^	0.42 ± 0.01^de^	37.87 ± 0.82^d^
13	7.58	0.65	1.45 ± 0.09^de^	0.41 ± 0.01^e^	32.13 ± 0.26^g^

*Note:* Data are presented as mean ± SD (*n* = 3); ^a‐g^means with the same superscript in a column do not vary significantly (*p* < 0.05) from each other.

The ANOVA of the response surface model (Equations ((1), (2), (3))) is displayed. The *F*‐values for the models (i.e., Equations ((1), (2), (3))) were 5.01, 23.23, and 131.93, respectively, indicating that the models are significant. There is only a 0.01% chance that such high *F*‐values could occur due to noise. The pH and enzyme concentration significantly affect the response, as indicated by the *p*‐values being less than 0.05. The *R* ^2^ values indicate the degree to which the model was able to predict the response. The determination coefficients (*R* ^2^) of the models were 0.6257 for Equation (1), 0.9026 for Equation (2), and 0.9820 for Equation (3). When the *p*‐value is smaller than 0.05, the parameters are considerably varied, and the analysis result is statistically significant (Thakur et al. 2020). The model's significance and high correlation were demonstrated by the close agreement between predicted and observed values. Contour plots, derived from the RSM, provide a 2D projection of the reaction surface to predict outcomes. These plots illustrate variable interactions through their shapes: circular contours indicate weak or negligible interactions, while elliptical contours suggest stronger interactions between variables. This visual representation helps in understanding the complex relationships within the experimental system.

**FIGURE 2:**
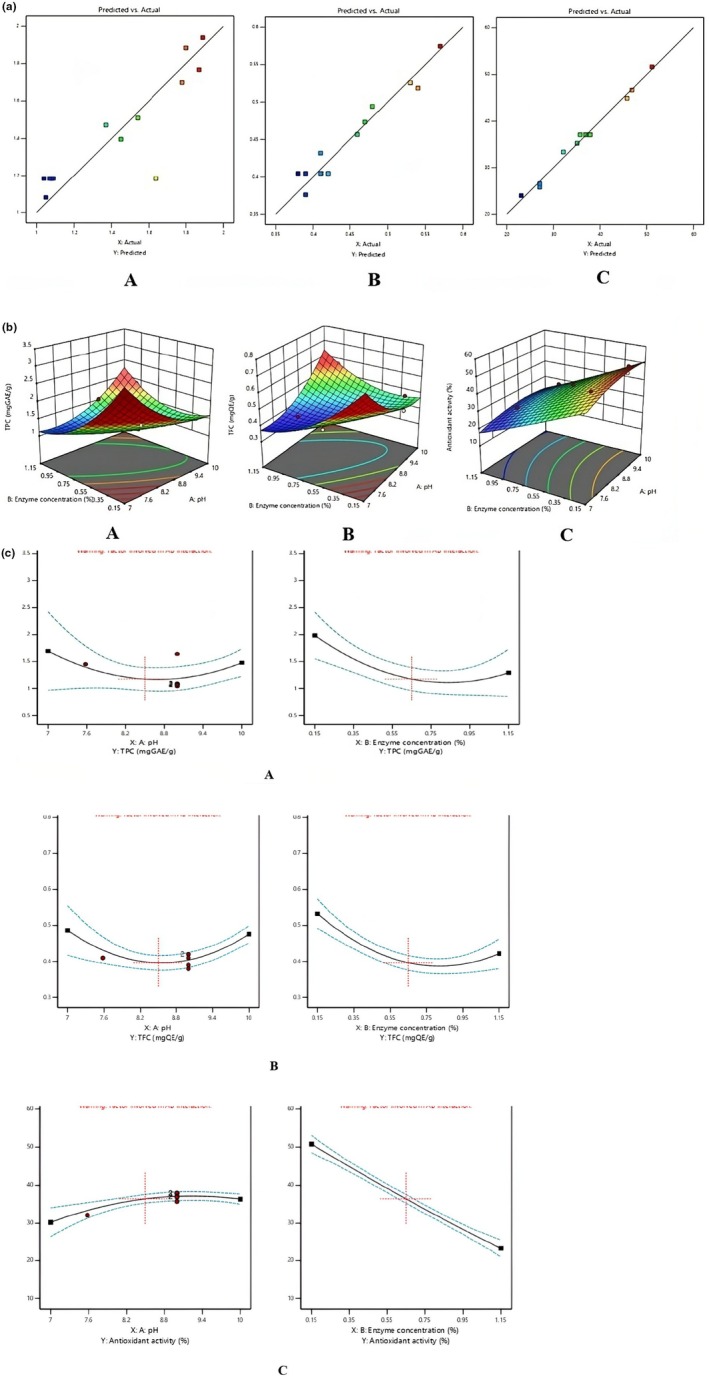
(a) Predicated versus actual graph of TPC (A), TFC (B), antioxidant activity (C). (b) Graphical representation for (A) TPC (mg GAE/g) between enzyme concentration (%) and pH; (B) TFC (mg QE/g) between enzyme concentration (%) and pH; (C) antioxidant activity between enzyme concentration (%) and pH. (c) Linear correlation between process variables; TPC (A), TFC (B), and antioxidant activity (C).

Figure 2a presents a linear graph of the predicted and experimental values for the elimination of non‐protein components. All points were correctly dispersed near the line, indicating a strong relationship between the expected and experimental data. Furthermore, Qasim et al. (2020) reported that the model would be more accurate if the data is closer to the reference line.

Figure 2b presents response surface plots, which three‐dimensional visual representations illustrate the combined effects of two variables on the reaction outcome while keeping the third variable constant at its center point. These plots provide a clear visual indication of how pairs of variables interactively influence the reaction. Specifically, Figure 2c shows the impact of pH and enzyme concentration on the TPC, TFC, and antioxidant activity of hydrolysates. Overall, it can be shown that the two variables have significant effects on the TPC, TFC, and antioxidant activity of the hydrolysate. The central points of the contour plots were used to identify the optimized conditions. The validation experiments were carried out at optimized conditions, which were at an enzyme concentration of 0.3% and pH 8, yielding an average TPC of 1.80 mg GAE/g, TFC of 0.54 mg QE/g, and antioxidant activity of 45.80%. The desirability graph with the two variables, enzyme concentration and pH, indicates a desirability of 1.000.

Figure 2c shows that as the pH and enzyme concentration increase, the TPC levels significantly decrease (*p* < 0.05). An alkaline pH causes the formation of phenolate ions, which are oxidized in the presence of oxygen to create semiquinone radicals, eventually leading to quinones and a decrease in overall TPC (Pasquet et al. 2024). Higher enzyme concentrations typically lead to a decrease in TPC because increased hydrolytic activity tends to break down phenolic compounds more extensively (Aenglong et al. 2024). The pH and enzyme concentration increase, whereas the TFC levels decrease significantly (*p* < 0.05) because the hydroxyl groups undergo deprotonation (Nayik et al. 2016). As enzyme concentration gradually increased, molecular competition occurred, inhibiting the transmission and release of the active ingredients. This inhibition prevented the dissolution of flavonoids, resulting in a decrease in the TPC levels (Yin et al. 2023). In Figure 2c, it shows that as pH increases, the antioxidant activity also increases significantly (*p* < 0.05). Similar results were found in a study by Pakbin et al. (2022), where the antioxidant activity increased with an increase in the pH of bovine collagen hydrolysate. It was also observed that enzyme concentration increases with a significant decrease in the antioxidant activity (*p* < 0.05). The results are comparable with the findings of Abduh et al. (2022), where the antioxidant activity of protein hydrolysate derived from the larvae of the black soldier fly decreased with an increase in enzyme concentration. Table 3b represents the Quadratic model for TPC, TFC, and antioxidant activity.

### 3.3 Techno‐Functional Properties of Protein Hydrolysate

#### 3.3.1 Bulk Density and Tapped Density

Bulk density and Tapped density of the hydrolysate are given in Table 4. The bulk density and tapped density were 0.51 and 0.66 g/mL, respectively, for the hydrolysate. The results were compared with the results of casein hydrolysate by Sarabandi et al. (2018) where the bulk density was 0.281 g/mL and tapped density was 0.374 g/mL. In another study by Zaitoun et al. (2022), the bulk density and tapped density of whey protein hydrolysate were found to be 0.33 and 0.45 g/mL. In comparison with whey and casein hydrolysate, soybean oil seed cake protein hydrolysate has higher bulk density and tapped density. Bulk density and tapped density are crucial parameters in understanding and predicting the behavior of powders. Bulk density plays a vital role in characterizing powder flow properties. Density is a critical variable that guides the design of processes involving volumetric or gravimetric handling of materials, such as those where a specific mass of powder needs to be compacted into a final product form (Vasilenko et al. 2013).

**TABLE 4:** Functional properties of soybean oil seed cake protein hydrolysate.

Functional property	Values
Bulk density (g/mL)	0.51 ± 0.01
Tapped density (g/mL)	0.66 ± 0.01
Foam capacity (%)	22.00 ± 1.0
Foam stability (%)	50.40 ± 0.52
Water absorption index (g/g)	2.28 ± 0.07
Water solubility index (%)	59.66 ± 1.52
Oil absorption capacity (g oil/g sample)	1.34 ± 0.34

*Note:* Data are presented as mean ± SD (*n* = 3).

#### 3.3.2 Foam Capacity and Foam Stability

The foam capacity and foam stability of the hydrolysate are given in Table 4. Foam Capacity and foam stability were 22% and 50.40%, respectively, for the hydrolysate. The results were compared with the results of protein hydrolysates from defatted *Camellia oleifera* seed cake, where the foam capacity and foam stability were seen as 28% and 45.7%, respectively. Protein is the major component influencing foaming capacity. The high protein content in the hydrolysate enhances its foaming capacity and foam stability. In the food industry, foams play a crucial role in creating foam‐based products like whipped toppings, mousses, meringues, and aerated baked goods (Amagliani et al. 2021).

#### 3.3.3 Water Absorption Index and Water Solubility Index

The water absorption index and water solubility index of the hydrolysate are given in Table 4. The water absorption index and water solubility index of the hydrolysate powder were found to be 2.28 (g/g) and 59.66% respectively. The results were compared with the results of protein hydrolysates from waxy hydrolyzed starch by Murúa‐Pagola et al. (2009), where the water absorption index and water solubility index were seen as 2.43 (g/g) and 3.6% respectively. The presence of carbohydrates and proteins, which are major components, contributes to a higher water absorption index, whereas a high lipid content tends to lower the water absorption index (Kaushal et al. 2012).

#### 3.3.4 Oil Absorption Capacity

The oil absorption capacity of the hydrolysate powder is given in Table 4. The oil absorption capacity of the hydrolysate powder was found to be 1.34 (g/g). The results found in a study by Vioque et al. (2000) showed that the oil absorption capacity ranged from 1.37 to 1.55(g/g), depending on the different degrees of hydrolysis for Rapeseed Protein Hydrolysates. In another study, the oil absorption capacity was 1.864 (g/g) for Pumpkin Oil Cake Globulin Hydrolysates. Higher porosity and surface area generally lead to higher oil absorption capacities (Meng et al. 2014).

### 3.4 Characterization Techniques

#### 3.4.1 Fourier Transform Infrared Spectroscopy (FTIR)

**FIGURE 3:**
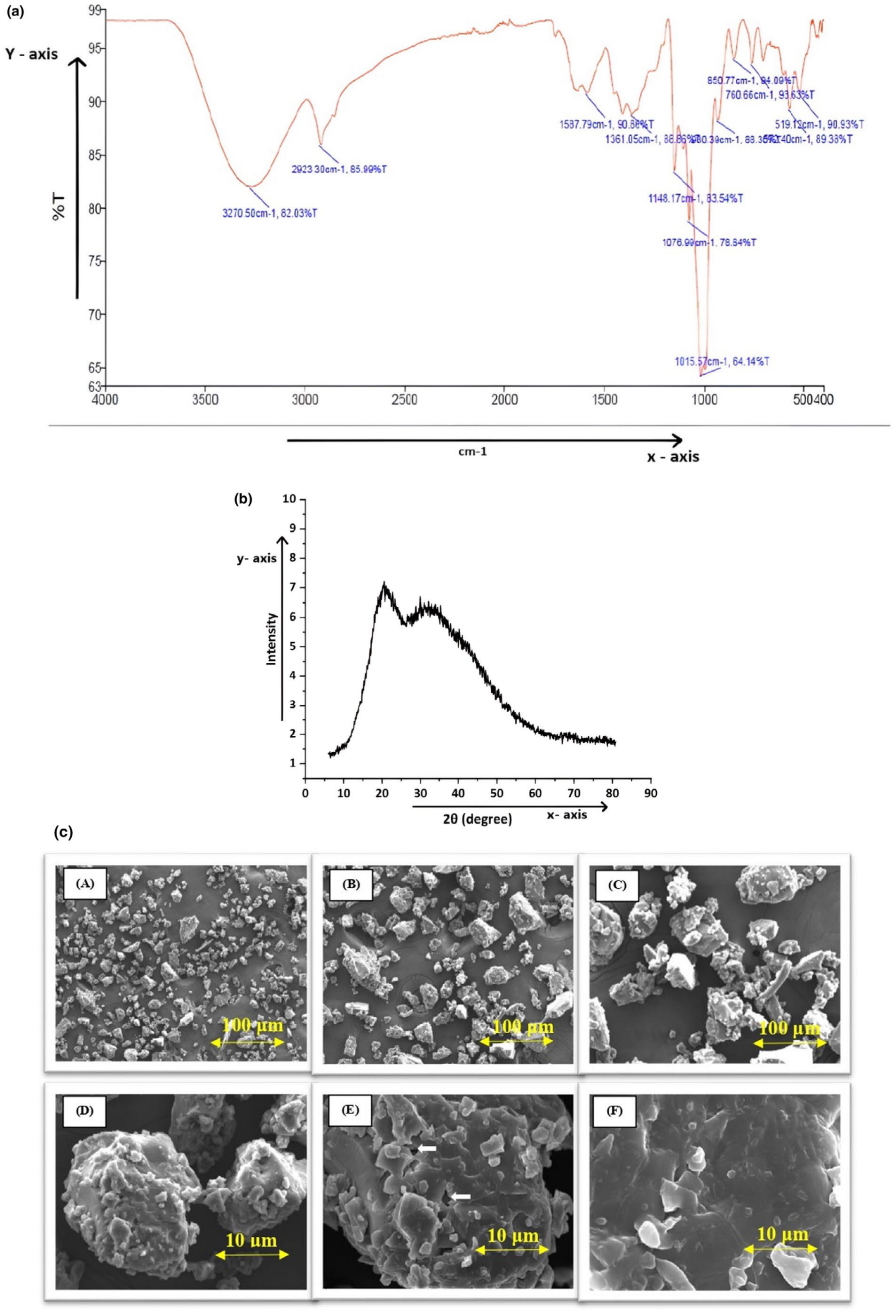
(a) FTIR spectra of hydrosylate. (b) X‐ray diffraction patterns of hydrosylate. (c) SEM micrographs of freeze‐dried protein hydrosylate powder. (A) 50×, scale bar: 100 μm; (B) 100×, scale bar: 100 μm; (C) 250×, scale bar: 100 μm; (D) 500×, scale bar: 100 μm; (E) 1000×, scale bar: 100 μm; and (F) 2500×, scale bar: 100 μm.

Figure 3a shows the result of FTIR spectra of hydrolysate prepared from soybean oil seed cake. The FTIR analysis of hydrolysate conducted within the spectrum range of 4000–400 cm^−1^ revealed the presence of various functional groups such as alcohols, alkanes, amines, fluoro compounds, and halo compounds (Wang et al. 2024). This indicates that the hydrolysate contains diverse organic acid functional groups. From the results, FTIR spectroscopy absorption at 3270.50 cm^−1^ revealed strong and broad stretched OH bonds, which belong to the alcoholic class; the alkane class at absorption 2923.30 cm^−1^ displayed medium‐intensity C–H stretching; the amine class at absorption 1587.79 cm^−1^ displayed medium‐intensity N–H bending; and alcohol (O–H bending) at absorption 1361.05 cm^−1^ displayed medium‐intensity alcohol (Nnamezie et al. 2021; Mechmeche et al. 2017; Kshirsagar et al. 2015). Tertiary alcohol (C–O stretching) was strongly absorbed at 1148.17 cm^−1^, primary alcohol (C–O stretching) was strongly absorbed at 1076.99 cm^−1^, fluoro compound stretching vibration was strongly absorbed at 1015.57 cm^−1^, halo compound (C–Cl stretching) was strongly absorbed at 760.66 cm^−1^, and halo compound (C–Br stretching) was strongly absorbed at 572.40 cm^−1^ (Priatni et al. 2020; Nnamezie et al. 2021).

#### 3.4.2 X‐Ray Diffraction (XRD)

Figure 3b shows the result of XRD of hydrolysate prepared from soybean oil seed cake. The hydrolysate shows a semi‐crystalline nature as confirmed by the nature of the peaks. The major peaks observed at 2θ values are 20.36°, 29.90°, and 28.80°, which indicate its crystalline characteristic. Meanwhile, the broad peaks at the 2θ region, which were seen as 33.88°, 35.344°, and 40.20°, suggest that the protein hydrolysate sample does not contain a significant amount of highly crystalline protein phases and has a largely amorphous structure (Yuan et al. 2023). These could be related to mineral phases like oxides, phosphates, or sulfates that sometimes occur naturally in plant‐based protein sources like soybeans (Du 2024). Similar findings were reported by Erciyes and Ocak (2019) in their study of collagen hydrolysate films.

#### 3.4.3 Field Emission Scanning Electron Microscopy (FESEM)

The study focused on examining the morphological characteristics of protein hydrolysate powder at various magnification levels: (A) 50×, (B) 100×, (C) 250×, (D) 500×, (E) 1000×, and (F) 2.500×. The FE‐SEM (Field Emission Scanning Electron Microscope) analysis revealed that the freeze‐dried protein hydrolysate powder exhibited a broken, brittle nature and possessed a microporous structure, as depicted in Figure 3c. The observed morphological characteristics and structural features of the hydrolysate can be attributed to the influence exerted by the specific type of dryer employed during the drying process, which ultimately shaped the structure and morphology of the particles (Akbarbaglu et al. 2019). The observed structure of the hydrolysate as broken and brittle is potentially a consequence of the elevated moisture content present, coupled with the incapability of the drying technique employed to facilitate the formation of spherical particle morphologies (Kumar et al. 2021). Similar results were found for soybean hydrolysate in a study by Wang et al. (2020) where the hydrolysates had irregular and wrinkled surfaces.

### 3.5 Lipase Inhibition Assay

The lipase inhibition activity of the hydrolysate powder was found to be 40.33%. The results found in a study by Ketprayoon et al. (2021) showed that the lipase inhibition activity of de‐oiled rice bran hydrolysate was 19.25% with a molecular weight of 5–10 kDA. Recent research by Ngoh et al. (2017) has shown that synthetic sequences derived from pinto bean peptides were able to inhibit lipase activity, with inhibition levels ranging from 23% to, as high as, 87%. Protein hydrolysates generally have improved solubility and bioavailability compared to intact proteins. This increased solubility and bioavailability can facilitate the interaction of the peptides with lipase enzymes, leading to better inhibitory effects (Abeer et al. 2021).

### 3.6 Amylase Inhibition Assay

The amylase inhibition activity of the hydrolysate powder was found to be 53.47%. The results were compared with the results of protein hydrolysates from Seaweed Protein Alcalase hydrolysate, which were 31.73% (Admassu et al. 2018). Another study by Sarteshnizi et al. (2021) showed results of 41.47% amylase inhibition activity for Papain hydrolysate. High amylase inhibitory activity in protein hydrolysates is due to the presence of bioactive peptides with specific amino acid sequences that interact strongly with the active site of the enzyme, competitively inhibiting its activity (Baba et al. 2021).

### 3.7 Glucose Uptake Assays Using Dialysis Membrane

**FIGURE 4:**
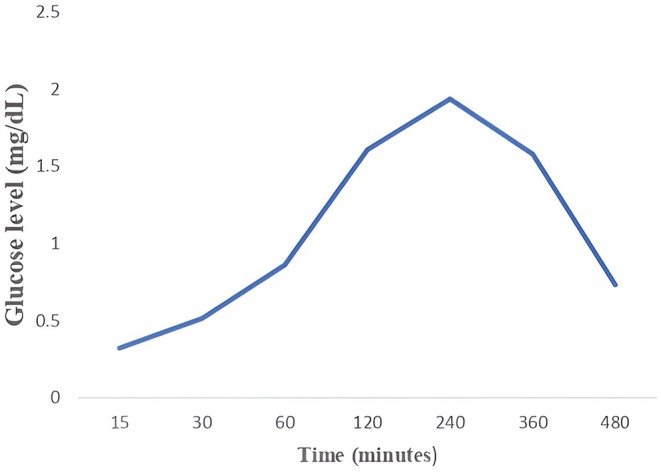
Glucose level (mg/dL) of hydrosylate.

The results of the glucose levels for the protein hydrolysate are shown in Figure 4. Glucose levels were measured at various intervals of time (15, 30, 60, 90, 180, 240, 360, and 480 min). The highest glucose retention time (1.939 mg/dL) was seen at 240 min. Furthermore, the glucose retention decreased at 360 min in the protein hydrolysate. Protein hydrolysates have been found to exhibit their antidiabetic effects through various mechanisms, including the inhibition of digestive enzymes and Dipeptidyl peptidase IV (DPP‐IV), as well as reducing blood glucose levels and enhancing insulin uptake (Kehinde and Sharma 2018).

## 4 Conclusion

This study successfully optimized the hydrolysis conditions for soybean oil seed cake using RSM to produce a protein hydrolysate with enhanced nutritional and functional properties. The optimized hydrolysate powder contained 60.33% protein, significantly higher than the 46.26% in the original oil seed cake. Optimal hydrolysis conditions were identified as pH 8 and 0.3% enzyme concentration. Under these conditions, the hydrolysate exhibited superior antioxidant activity (45.80%), total phenolic content (1.80 mg GAE/g), and total flavonoid content (0.54 mg QE/g) compared to other experimental runs. The hydrolysate also demonstrated improved techno‐functional properties, including bulk density, tapped density, foaming capacity, foam stability, water absorption index, water solubility, and oil absorption capacity, surpassing those reported for other protein hydrolysates in the literature. Characterization techniques provided insights into the hydrolysate's structural and morphological features. FTIR analysis revealed diverse organic acid functional groups, while XRD analysis indicated a semi‐crystalline nature. SEM imaging showed a microporous, broken, and brittle morphology, attributable to the drying technique used. The optimized hydrolysate exhibited promising bioactivities, including lipase inhibition (40.33%), amylase inhibition (53.47%), and prolonged glucose retention time up to 240 min in an in vitro model. These bioactivities suggest potential applications in managing obesity, diabetes, and related metabolic disorders. Overall, the optimized soybean oil seed cake protein hydrolysate proves to be a valuable product with diverse applications in the food, pharmaceutical, and biotechnology industries. Its high protein content, superior functional properties, and bioactive potential make it a promising ingredient for developing functional foods, nutraceuticals, and other value‐added products. Further studies are recommended to explore the specific mechanisms underlying the observed bioactivities and evaluate the hydrolysate's performance in various product formulations and industrial applications. Despite the promising functional and bioactive properties of the soybean oil seed cake protein hydrolysate, several limitations should be acknowledged. The study was conducted under in vitro conditions, and in vivo validation is necessary to confirm bioavailability and efficacy. Additionally, the scalability of the hydrolysis process for industrial production was not addressed. The economic feasibility and long‐term storage stability of the hydrolysate remain unexplored. Further studies are required to evaluate sensory attributes and application performance in real food systems.

## Author Contributions

**Haifa Hamza:** resources (equal), writing – original draft (equal). **Deepika Kaushik:** conceptualization (equal), data curation (equal), formal analysis (equal), investigation (equal), methodology (equal), resources (equal), software (equal), validation (equal), visualization (equal), writing – original draft (equal), writing – review and editing (equal). **Harmandeep Kaur:** software (equal), writing – review and editing (equal). **Rajdeep Kaur:** resources (equal). **Yassine Jaouhari:** data curation (equal), formal analysis (equal), writing – review and editing (equal). **Charalampos Proestos:** resources (equal), software (equal), writing – review and editing (equal). **Mukhtar Ahmed:** resources (equal), software (equal), writing – review and editing (equal). **Mohammad Rizwan Khan:** resources (equal), software (equal), writing – review and editing (equal). **Fatih Oz:** data curation (equal), formal analysis (equal), writing – review and editing (equal). **Mukul Kumar:** conceptualization (equal), data curation (equal), formal analysis (equal), funding acquisition (equal), investigation (equal), methodology (equal), project administration (equal), resources (equal), software (equal), supervision (equal). **Matteo Bordiga:** writing – review and editing, Resources.

## Conflicts of Interest

The authors declare no conflicts of interest.

## Data Availability

The data that support the findings of this study are available from the corresponding author upon reasonable request.
